# Autosomal Dominant Polycystic Kidney Disease: From Pathogenesis to Organoid Disease Models

**DOI:** 10.3390/biomedicines13071766

**Published:** 2025-07-18

**Authors:** Alexandru Scarlat, Susanna Tomasoni, Piera Trionfini

**Affiliations:** Istituto di Ricerche Farmacologiche Mario Negri IRCCS, Centro Anna Maria Astori, Science and Technology Park Kilometro Rosso, 24126 Bergamo, Italy; alexandru.scarlat@marionegri.it (A.S.); susanna.tomasoni@marionegri.it (S.T.)

**Keywords:** ADPKD, hPSCs, organoids, disease modelling

## Abstract

Mutations in *PKD1* and *PKD2* cause autosomal dominant polycystic kidney disease (ADPKD), the most common renal genetic disease, leading to the dysregulation of renal tubules and the development of cystic growth that compromises kidney function. Despite significant advances in recent decades, there remains a considerable unmet clinical need, as current therapeutics are not effective at slowing or halting disease progression. Although preclinical animal models have been used extensively, the translatability of such findings is uncertain and human-relevant disease models are urgently needed. The advent of pluripotent stem cells (PSCs) and their ability to more accurately recapitulate organ architecture and function has allowed for the study of renal disease in a more physiological and human-relevant setting. To date, many research groups have studied ADPKD using PSC-derived kidney organoids, identifying many dysregulated pathways and screening drug candidates that may yield effective therapies in the clinic. In this review article, we discuss in detail the development of PSC-derived kidney organoids as ADPKD models and how they have advanced our understanding of the disease’s pathogenesis, as well as their limitations and potential strategies to address them.

## 1. Introduction

Autosomal dominant polycystic kidney disease (ADPKD) is the single most common genetic cause of chronic kidney disease (CKD), and it is estimated to cause up to 10% of cases of end-stage renal disease (ESRD) [1,2]. In affected patients, cysts emerge from renal tubules, leading to a significant increase in total kidney volume (TKV), paralleled by a progressive loss in renal function, which inevitably causes ESRD [3]. While early studies on ADPKD incidence indicated a prevalence ranging from 1 in 400 to 1 in 1000 individuals [4,5], a recent meta-analysis of eight European epidemiological studies determined an incidence of 2.7 in 10,000 individuals [6]. Renal cyst formation is the main characteristic of ADPKD, and patients are frequently affected by episodes of gross haematuria, cyst infections, and cysts bursting, as well as nephrolithiasis [7,8,9]. Additionally, numerous extrarenal manifestations are also common, including cardiovascular dysfunction [10,11], intracranial aneurysms [12], and cysts in the liver and pancreas [13,14]. Importantly, cardiovascular events are a leading cause of death in ADPKD patients [15,16], and hypertension (HT) may be a major contributing factor, as it presents in almost 50% of patients before any loss in renal function, and in the late-stage patients, almost all are affected [17].

## 2. Genetics

Patients with ADKPD generally have mutations in the genes *PKD1* (encoding polycystin 1, PC1) and *PKD2* (encoding polycystin 2, PC2), accounting for 78% and 15% of cases, respectively [18]. *PKD1* encodes a transcript of 14 kb in size, contains 46 exons, is located on chromosome 16p13.3, and was first identified in 1994 [19,20]. *PKD2* encodes a transcript of 5.4 kb, contains 15 exons, is located on chromosome 14q21-23, and was sequenced and characterised in 1996 [21]. Patients with mutations in *PKD1* have been shown to have a significantly worse prognosis than those with *PKD2* mutations, as they tend to progress to ESRD approximately 20 years earlier [22]. Interestingly, among patients with *PKD1* mutations, there is also considerable variability in disease progression, as truncating mutations cause ESRD about 10 years earlier than non-truncating mutations [23].

Patients with ADPKD possess heterozygous mutations, generally in *PKD1* or *PKD2*, and in humans, they appear sufficient to lead to the formation of severe cystic disease during adult life. Numerous studies in mice have shown that biallelic loss-of-function mutations in either the *Pkd1* or *Pkd2* homologues cause embryonic lethality. Importantly, it seems that this effect is caused not only by severe defects in renal development but also by the occurrence of pancreatic ductal cysts, pulmonary hypoplasia, and myocardial and endothelial dysfunction [24,25,26], possibly indicating that homozygous mutations may also cause embryonic death in humans. Moreover, *Pkd1* and *Pkd2* seem to be essential in placental formation, possibly contributing to the essential role of the polycystins in embryonic development [27]. A study in cynomolgus monkeys similarly showed that the near-complete deletion of *PKD1* is lethal perinatally due to a number of congenital defects, including severe renal cystic disease and pulmonary hypoplasia [28]. Interestingly, this study showed that monkeys with monoallelic *PKD1* mutations slowly developed cysts later in life, similar to ADPKD in humans. It seems that this is similar to the role of PC1 in humans, as a study reported that parents with ADPKD lost multiple foetuses during late gestation, possibly due to homozygous *PKD1* mutations inherited from both parents [29]. A case report also described a neonate with biallelic *PKD1* mutations with a severe renal phenotype [30].

Studies in mice have shown that *Pkd1* heterozygous knockout has a mild phenotype, while mice with an ~80% loss of *Pkd1* transcript due to a hypomorphic allele develop severe cystic disease [31]. Additionally, the timing of *Pkd1* disruption has a dramatic impact on cyst formation, as early knockout causes significantly more severe disease than gene disruption in adult mice [32]. These findings also indicate that there is a critical threshold for *Pkd1* activity, below which cyst formation is initiated, and possibly that the timing of the loss of polycystin activity may account for some of the variability seen in disease progression in ADPKD patients. Recently, a mouse model with mosaic loss of *Pkd1* has been developed, where ~8% of cells undergo mutation, and these mice develop cystic disease slowly and more closely mimic the progressive and exponential cystic growth seen in humans [33,34]. Similarly, the aforementioned study in monkeys showed that the mutation rate correlated strongly with the degree of cystic disease in animals with mosaic *PKD1* deletion [28]. This would therefore seem to indicate that only a small proportion of cells need to lose PC1 expression to trigger slow and progressive renal disease. Furthermore, although cells with *PKD1/Pkd1* loss may begin the initial cystogenesis, evidence in these studies suggests that paracrine effects may induce the dysregulation of wild-type cells and trigger further cystogenesis in a cascading manner [28,34].

Early studies on ADPKD patients have revealed that in a small proportion of cysts, loss of heterozygosity (LOH) had taken place, meaning that the normal *PKD1* or *PKD2* allele was disrupted [35,36,37,38]. Interestingly, the mutations in the normal allele varied to a considerable extent, indicating that there are no mutational hotspots in *PKD1* and *PKD2*, but instead that these genomic loci are highly unstable. This led to the formation of the “two-hit” hypothesis, which states that cyst initiation occurs when the normal allele of either *PKD1* or *PKD2* undergoes a mutation, as those tubular epithelial cells become dysfunctional and gain a growth advantage [39]. While compelling, the “two-hit” hypothesis could not initially explain why only a few cysts appeared to have undergone LOH. However, advances in sequencing technologies have overcome the challenges associated with sequencing *PKD1* and distinguishing it from its six highly homologous pseudogenes. A recent study utilised whole-genome sequencing (WGS) to determine whether the normal allele in ADPKD patients did indeed undergo somatic genetic alterations in cyst-lining cells [40]. The investigators found that in all patients studied, there were mutations in the normal allele and a total of 93% of cysts carried such alterations, providing strong evidence for the idea that the “two-hit” hypothesis is the main driver of ADPKD. This evidence therefore indicates that although ADPKD is an autosomal dominant disease at the phenotypic level, it may indeed be recessive at the cellular level.

## 3. Molecular Mechanisms

Numerous studies have implicated a multitude of molecular pathways that may contribute to ADPKD cyst initiation and progression, including cyclic adenosine monophosphate (cAMP), G-protein signalling, mechanistic target of rapamycin (mTOR), mitogen-activated protein kinase (MAPK), and Wingless-related integration site (Wnt) [41].

### 3.1. cAMP Signalling

Considering the fact that the PC1–PC2 complex is hypothesised to act principally as a Ca^2+^ channel in the cilium, and possibly other cellular compartments, Ca^2+^ signalling has received significant attention. In numerous settings, it has been shown that *PKD1* or *PKD2* mutations lead to dysregulated Ca^2+^ signalling and a loss of the mechanosensory properties of the PC1-PC2 complex [42,43]. Cyst expansion and proliferation can be accelerated by increasing cAMP concentrations [44], and increased cAMP has been observed in animal models [45]. This is important, as adenylyl cyclases (ACs), which synthesise cAMP, such as AC5 and AC6, are directly inhibited by higher Ca^2+^ concentrations, and in fact, the disruption of either AC5 and AC6 is known to slow ADPKD progression in mouse models [46,47]. Phosphodiesterases (PDEs) are enzymes that hydrolyse cAMP; the expression and levels of PDE1, the only calcium-inhabitable PDE, have been shown to be decreased in ADPKD models [48], and its disruption exacerbates cyst progression and increased cAMP target gene expression [49]. Further, the disruption of PC2 in cilia causes the activation of AC6 [50]. cAMP is thought to promote disease progression by stimulating proliferation via Ras/Raf-extracellular signalling kinase (ERK) pathways [51] and fluid secretion via cystic fibrosis transmembrane conductance regulator (CFTR) [52].

### 3.2. G-Protein Signalling

As expected from its structure, PC1 interacts with G-proteins, similarly to conventional G protein-coupled receptors (GPCRs), and Parnell and colleagues first reported that PC1 interacts with Gα_i_ and Gα_o_ proteins [53]. The interaction of PC1 with G-proteins regulates numerous pathways that have been implicated in ADPKD, including cell survival and proliferation via phosphatidylinositol 3- kinase (PI3K)–Akt and c-Jun N-terminal kinase (JNK)–activator protein 1 (AP1) [54,55], as well as Ca^2+^ signalling via phospholipase C and the activation of nuclear factor of activated T cells (NFAT) [56]. Interestingly, it has been shown that when assembled in a complex with PC2, PC1 is prevented from interacting with G-proteins, indicating that this regulatory effect may be lost in ADPKD, leading to dysregulated G-protein signalling [57]. Therefore, it may be that G-protein activation promotes cystogenesis in the absence of PC2. Alternatively, Delmas and colleagues showed that using an activating antibody against PC1 could simultaneously activate G-proteins, as well as PC2-mediated increases in Ca^2+^ [58]. Additionally, there is evidence that the loss of PC1 causes a dysregulated balance between G_α_ and G_β/γ_ signalling as a novel mutation at the G-protein binding site of *PKD1,* preventing signalling via G-proteins, causing ADPKD [59,60]. This therefore paints a very complex picture, where in certain situations, PC1 is thought to promote pathways involved in ADPKD, while in others, its loss is detrimental and causes cystic disease.

### 3.3. mTOR Signalling

The mTOR signalling pathway is known to affect cell growth, as well as numerous metabolic processes, including protein and lipid synthesis, as well as autophagy [61]. The central core of the mTOR pathway consists of the mTOR complex 1 (mTORC1) and mTORC2, which are known to have several divergent functions based on their binding partners. mTORC1, for instance, can activate p70 S kinase 1 (S6K1) to promote mRNA translation [62], while mTORC2 binds and phosphorylates AGC kinases such as protein kinase C (PKC) and Akt [63]. The tuberous sclerosis complex (TSC) is the main regulator of mTORC1/2 [64], and it in turn can be inhibited by pathways such as protein kinase B (PKB)/Akt [65]. PC1 directly interacts with TSC2 and inhibits mTOR signalling, and active mTOR signalling has been found in cyst-lining cells in patients and mouse models, while mTOR inhibition reduces cysts in mice [66,67].

### 3.4. Wnt Signalling

Wnt signalling has also been implicated in the initiation of cystogenesis in ADPKD. Wnt signalling can be divided into Wnt-β-catenin (canonical) and Wnt-planar cell polarity (PCP) signalling, and there is evidence that both these pathways may play a role in ADPKD [68]. Constitutive Wnt-β-catenin signalling appears to cause severe cystic disease [69], and mutations in *PKD1* or *PKD2* cause canonical Wnt pathway activation [70,71]. The effect of canonical Wnt inhibition in ADPKD seems unclear, as studies in mice have yielded conflicting results, showing that it can both ameliorate and exacerbate cystogenesis and disease progression [72,73]. The PCP pathway has also been involved in cystogenesis, as the loss of orientated cell division (OCD) appears to play an important role in tubular dilation [74], although the loss of OCD may be a feature of ADPKD, taking place after cyst formation has begun, and it is not sufficient on its own to initiate cystogenesis [75]. The role of Wnt signalling in ADPKD therefore remains controversial and it is unclear whether its pharmacological modulation could be therapeutic.

### 3.5. Fluid Secretion

Cysts in ADPKD arise from renal tubules that dilate and eventually separate from the original tubule, subsequently expanding by fluid secretion. They have been categorised into “null-gradient” cysts, which have no transepithelial resistance, and “gradient cysts”, which possess transepithelial resistance, indicating that transport mechanisms across a tight epithelial barrier maintain an electrical potential [76]. Cyst fluid secretion has been reported to be dependent on the Na^+^-K^+^-ATPase, which is mislocalised to the apical membrane of cysts, rather than basolaterally [77]. Importantly, fluid secretion is generally driven by Cl^-^ transport, and cysts have been found to be lumen-electronegative [78]. Evidence in renal cells also indicates that cAMP stimulation promotes Cl^−^—driven fluid secretion, specifically by CFTR, leading to cyst expansion [79,80]. Mechanistically, cAMP-activated protein kinase A (PKA) is known to activate CFTR, thus linking increased cAMP in ADPKD with cyst progression [81]. Consequently, CFTR inhibitors have been shown to slow cystogenesis [82]. Recently, anoctamin 1 (ANO1, also known as TMEM16A) has been identified as a Ca^2+^-activated Cl^-^ channel that promotes fluid secretion in *PKD1* or *PKD2* knockout models, and its pharmacological inhibition slows cyst growth in cell cultures, as well as in mice [83,84].

## 4. Pharmacological Treatment

Tolvaptan is an inhibitor of the arginine vasopressin V2 receptor (AVPR2) in the renal collecting duct (CD). Arginine vasopressin (AVP) is a circulating hormone acting on vasopressin receptors that regulates fluid volume in response to low volume or high osmolarity by stimulating increased water reabsorption from renal tubules [85]. In ADPKD, AVPR2 activation has been shown to stimulate ACs, thus increasing the cAMP concentration and promoting disease progression [86]. Tolvaptan is currently the only drug approved by the Federal Drug Administration (FDA) and the European Medicines Agency (EMA), and its approval was based on the results of two large phase 3 clinical trials. The TEMPO 3:4 trial recruited 1445 ADPKD patients and showed that Tolvaptan slowed the increase in TKV by 50% and the loss of renal function by 30% [87]. The REPRISE trial investigated Tolvaptan use in 1370 patients with early- and late-stage ADPKD and showed a reduction in loss of renal function by 35% [88]. Despite these very promising results, Tolvaptan has only been approved for the treatment of rapidly progressing late-stage ADPKD [89] due to its associated side effects. Patients generally suffer from polyuria and polydipsia, which are caused by the mechanism of action of Tolvaptan and may affect compliance with treatment. Hepatotoxicity is a significant idiosyncratic side effect associated with Tolvaptan use that has been observed in up to 5% of patients, and it is for the most part reversible upon treatment discontinuation [90]. Considering that Tolvaptan has a modest effect on disease progression and a poor side effect profile, limiting its use to only rapidly progressing patients, there is an urgent requirement for additional drugs for ADPKD.

Somatostatin is a peptide hormone that acts on somatostatin receptors (SSTRs) and has a multitude of pleiotropic effects, including the inhibition of ACs [91]. Given its mode of action in counteracting cAMP signalling, the activation of SSTRs has been proposed to be effective in ADPKD [92]. Multiple somatostatin analogues such as octreotide and pasireotide have been shown to be effective in animal models [93,94]. Octreotide long-acting release (LAR) has been approved in Italy by a special decree (n. 1264, 3 August 2018) based on findings from the ALADIN-2 trial [95]. The study found that Octreotide-LAR significantly slowed the increase in TKV, and, while the loss of renal function was not significantly affected, fewer patients on Octreotide-LAR progressed to ESRD. A recent study in a small cohort of patients with early-stage ADPKD showed that a combination treatment of Tolvaptan and Octreotide-LAR ameliorated compensatory hyperfiltration and significantly decreased TKV compared to Tolvaptan treatment alone [96]. Octreotide-LAR has, however, been shown to significantly reduce the total liver volume (TLV) in patients with hepatic cysts [97,98]. A clinical trial investigating another somatostatin analogue, Pasireotide-LAR, showed that it was effective in decreasing both TKV and TLV; however, similarly to Octreotide-LAR, there was no effect on renal function loss [99]. 

Given that treatment for ADPKD is very limited, there have been efforts to develop new drug targets. Despite the apparent role of mTOR signalling in ADPKD uncovered using animal models, numerous clinical trials have failed to show a beneficial effects in terms of cyst size or renal function [100,101,102]. A study in mice suggested that these findings may be due to the dysregulation of mTOR-PI3K-ERK negative feedback loops [103]. This, however, is incongruent with the extensive evidence supporting the beneficial effects of mTOR inhibition in animal models [67,104], indicating that there may be interspecies differences accounting for this effect. β3 adrenergic receptors (β3-ARs) stimulate cAMP in renal tubules, and in a mouse ADPKD model, their inhibition was shown to decrease cAMP levels and ameliorate cystogenesis [105]. Prostaglandin E2 (PGE2) has also been identified as a cAMP stimulator, acting on PGE2 receptors (EP) receptors, and a study has shown that the cilia–specific activation of EP4 receptors promotes cystogenesis [106]. Although numerous studies have implicated PGE2 in the progression of cysts, it would appear that the inhibition of EP receptors may activate an inflammatory mechanism that exacerbates the disease phenotype [107]. AMP-stimulated protein kinase (AMPK) is a protein kinase that can inhibit CFTR and mTORC1, as well as cAMP, by activating PDE4B [108,109]. Metformin can indirectly activate AMPK, and two small clinical trials have shown that there are no significant safety concerns; however, a high proportion of patients were unable to tolerate the maximal dose [110,111]. A phase 3 trial in now underway to evaluate the efficacy of metformin in treating ADPKD (NCT04939935). For a deeper discussion on drugs currently under preclinical and clinical development, readers are directed to more extensive reviews [112,113].

## 5. Embryonic Kidney Development

The renal parenchyma of the mammalian metanephric kidney is composed of nephrons, epithelial tubules that function to filter blood and excrete waste. The epithelial segment of the kidney is formed by the interaction of the ureteric bud (UB) with the metanephric mesenchyme (MM), with the former generating the CD and the latter forming the glomerulus, proximal tubules, loop of Henle, and distal and connecting tubules [114]. While both the UB and MM are derived from the mesoderm lineage following gastrulation, extensive lineage-tracing studies in mice have revealed that their progenitors diverge early on from the primitive streak stage and require different extracellular signals [115,116]. Progenitors of the MM have been shown to remain caudally at the primitive streak for a longer period of time, migrating rostrally later than the UB progenitors, forming first the posterior intermediate mesoderm and then the MM [115]. UB progenitors, on the other hand, leave the primitive streak earlier, forming the anterior intermediate mesoderm during their rostral migration. These cells then coalesce into an epithelial tubule called the nephric duct that migrates caudally and later fuses with the cloaca [116]. During its caudal elongation, the nephric duct approaches the MM, which secretes growth factors including glial cell-derived neurotrophic factor (GDNF), attracting cells in the epithelial tubule. This leads to the formation of the UB, which begins to migrate and invade the MM. The UB tubule then begins to branch dichotomously and to induce the surrounding MM to begin nephrogenesis, while the MM itself secretes growth factors that support UB branching [114]. While the epithelial segments of the kidney are derived from the UB and MM, stromal cells also play an essential role in correct organ patterning and morphogenesis, as well as being progenitors for a number of cell types, such as interstitial fibroblasts and mesangial cells [117].

## 6. Generating Renal Tissues from Pluripotent Stem Cells

Embryonic stem cells (ESCs) are a population found in the inner cell mass of the preimplantation blastocyst that subsequently differentiate into all the cell lineages found in the adult organism. The generation of human induced pluripotent stem cells (hiPSCs) from adult somatic cells has addressed the technical, as well as ethical, challenges associated with human ESCs (hESCs) and has paved the way for studying human disease in more physiological settings [118]. In this review, we will thereafter refer to hESCs and hiPSCs collectively as human pluripotent stem cells (hPSCs). In order to derive renal tissue from hPSCs, they must be guided through the same stages observed during embryonic development. As the developmental paths of the UB and MM have been shown to diverge early on, hPSC differentiation procedures must also reflect this. In order to generate organ-like structures containing the entire epithelial population, termed “kidney organoids”, hPSC-derived UB and MM must be generated separately and then combined [116]. More recently, the role of stromal cells has been increasingly recognised in guiding the differentiation of the epithelial component, as well as organising the higher-order structure and generating mesangial and renin-secreting cells [117,119]. In the last decade, numerous methodologies to generate renal tissue from hPSCs have emerged, serving as valuable tools in studying human development, as well as disease [115,116,120,121,122]. In this review, we focus on how advances in stem cell-derived organoid models in the last decade have advanced our understanding of ADPKD, as well as addressing their limitations and how these may be resolved. We exclusively discuss studies on kidney organoids generated from hPSCs used to model ADPKD and have excluded those using tubuloids generated from primary cells and work on other cystic kidney diseases.

## 7. Nephron Organoid ADPKD Models

**Figure 1:**
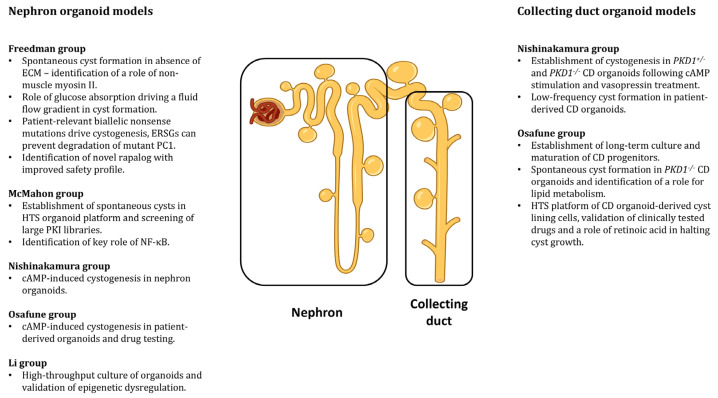
Overview of current hPSC-derived nephron and collecting duct organoid models that have been applied in the study of ADPKD. Kidney organoids have been increasingly used to recapitulate human cystogenesis and important disease processes, as well as to identify drug candidates. Figure generated using Biorender.

The first renal differentiation protocols from hPSCs generated nephron-like organoids with MM-derived cells, comprising glomerular podocytes and proximal and distal tubule-like cells [115,120,121]. Many research groups have developed nephron organoids derived from hPSCs, demonstrating their applicability in studying ADPKD (shown in Figure 1). Freedman and colleagues [123] generated epiblast spheroids from hPSCs in a Matrigel sandwich culture and then directed the differentiation towards renal organoids comprising glomerular podocytes and epithelial tubules. *PKD1^-/-^* and *PKD2^-/-^* cells were generated with CRISPR-Cas9 technology, and organoids generated from these cells differentiated normally, but upon extended culture, they were observed to spontaneously develop large cysts that grew continuously over months in the culture, although the cyst frequency was only 6% [123]. Subsequently, the same group then showed that dissecting ADPKD kidney organoids from the Matrigel extracellular matrix (ECM) significantly increased the rate of spontaneous cystogenesis up to 75% [124]. The cysts appeared to lose the phenotype of the original tubules that they were derived from, as they could no longer be identified as proximal or distal based on cell marker expression. *PKD2^-/-^* organoids also revealed that the loss of PC2 almost entirely abrogated the expression of PC1 at the protein, but not the mRNA, levels hinting at a post-translational mechanism [124]. A follow-up study then established an automated high-throughput culture method for kidney organoids to study cystogenesis and test drug compounds in ADPKD organoids [125]. As proof of concept, blebbistatin, an inhibitor of non-muscle myosin II, was found to significantly enhance cyst formation. ADPKD organoids cultured on this platform, however, showed a rate of cystogenesis of only 5–20%, a significant decrease from the previous rate of 75%, as organoids were cultured in the presence of ECM [124].

Fluid flow is important in maintaining and regulating the physiological function of renal epithelial cells and may be especially important in ADPKD, considering the potential role of the PC1-PC2 complex in mechanotransduction. The Freedman group employed a microfluidics device in a ADPKD organoid culture, determined that dynamic flow is sufficient to enhance cystogenesis, and discovered that this process is driven by absorption, rather than secretion [126]. Using a mouse model of ADPKD, they validated this absorptive mechanism in vivo, demonstrating the utility of organoids in studying disease processes. Nonsense mutations in *PKD1* and *PKD2* comprise up to half of the mutations in ADPKD patients, preventing the formation of full-length PC1 or PC2. Using CRISPR base-editing technology, the Freedman group generated patient-relevant *PKD1* and *PKD2* mutations in hPSCs and showed that while homozygous mutants readily formed large cysts, monoallelic mutants rarely developed cysts [127]. Ribosomal readthrough for premature stop codons using eukaryotic ribosomal selective glycosides (ERSGs) is under investigation for restoring CFTR activity in cystic fibrosis [128] and is safe in the clinic [129]. Treatment of ADPKD organoids with patient-relevant nonsense mutations restored PC1 and PC2 levels and prevented cyst formation, demonstrating the utility of organoids as models of human-specific disease features [127].

mTOR signalling has been identified as a core dysregulated pathway in ADPKD [67], and while mTOR inhibitors slow cyst growth in mice [104], clinical studies in humans have not yielded similar results and have demonstrated significant side effects, limiting the tolerated dose [101,102]. To address this shortcoming, the Freedman group recently used an established nephron organoid model and determined that AV457, an mTORC1-selective inhibitor, has a significantly better safety profile than everolimus, a non-selective mTOR inhibitor, while showing comparable efficacy at slowing cyst growth [130]. This study therefore serves as a proof-of-concept that human organoids can address the limitations of preclinical animal models and aid in the development of safe and effective therapeutics.

The Nishinakamura group took advantage of an hPSC-derived nephron organoid protocol to study cystogenesis in *PKD1^+/-^* and *PKD1^-/-^* tissues [131]. Organoids were efficiently generated from PC1-deficient cells, and upon extended exposure to forskolin, a cAMP agonist, they developed multiple large cysts, although control organoids also developed cysts, albeit to a lesser degree. The Osafune group took a similar approach, generating cyst-bearing *PKD1^+/-^* and *PKD1^-/-^* nephron organoids with the use of forskolin [132]. Cysts were also induced from ADPKD patient-derived organoids, and drugs such as mTOR and CFTR inhibitors were tested, demonstrating the validity of organoids in studying human disease. This study is significant, as it is the only one to date showing cyst formation in nephron organoids from a patient with *PKD1* mutations.

Tran and colleagues [133] took advantage of a previously published nephron organoid method [121] and aimed to upscale and automate culture conditions. Small organoids containing 1–2 nephrons were generated on microwell patterned plates, enabling spontaneous cyst formation in *PKD1^-/-^* and *PKD2^-/-^* organoids. A platform for automated imaging, coupled with the immobilisation of the organoids, was developed to monitor individual cyst growth and it was subsequently used to screen large libraries of protein kinase inhibitors, identifying potential drug candidates [133]. An important role of the Nuclear factor kappa B (NF-kB) signalling pathway was revealed, as its inhibition significantly slowed cyst growth, uncovering a potential new target. Recent studies in mice showed that inflammatory cytokine signalling was upregulated in the early phases [34], thus demonstrating the relevance of human organoids in disease modelling.

Huang and colleagues [134] established a method for the purification and expansion of nephron progenitor cells and subsequently established a high-throughput culture method for nephron organoids in a shaking culture. *PKD2^-/-^* organoids rapidly formed spontaneous cysts, and the efficacy of drugs targeting the epigenome was demonstrated, confirming the role of epigenetic dysregulation in cyst progression [135,136].

## 8. UB Organoid ADPKD Models

Considering that in ADPKD large, severe cysts are predominantly found in the renal CD [137,138], research groups have also employed hPSC-derived UB organoids for ADPKD modelling (shown in Figure 1). The Nishinakamura group generated *PKD1^-/-^PKD1^+/-^* UB organoids, showing that their differentiation was not adversely affected by PC1 loss, although no cysts were formed [131]. Treatment with forskolin for an extended time was sufficient to induce cystic-like growths from the stalks of *PKD1^+/-^* and *PKD1^-/-^* organoids; however, this was observed only in a small proportion of tubules. Cysts could also be induced with arginine vasopressin (AVP); however, the cyst frequency was very low, likely due to the lack of AVPR2 expression. Additionally, the origin of the cystic structures remained unclear, as they seemed to resemble small outgrowths from the tubular epithelia, rather than tubular dilations, as is seen in ADPKD patients. Finally, cysts were induced with forskolin in a patient-derived UB organoid, although the cyst frequency was low and the effect of AVP was not reported [131]. Despite this, this is the only report of cystogenesis in patient-derived UB organoids.

A study from the Osafune laboratory showed that the long-term expansion of UB progenitors is sufficient to enhance the maturity of CD organoids and that maturation is required for spontaneous cystogenesis in *PKD1^-/-^* organoids [139]. Importantly, all mature *PKD1^-/-^* organoids formed severe cysts, which were exacerbated by forskolin treatment, without the need to remove extracellular matrix [124], preserving the signals required for appropriate cell polarity. It was then revealed that the metabolism of *PKD1^-/-^* organoids was dysregulated at the level of cell cycle regulation and a role for dysregulated lipid metabolism was identified. Fluvastatin, an inhibitor of cholesterol production, was then shown to significantly reduce cyst formation [139]. A high-throughput culture method was also established using single cyst-lining cells, validating the efficacy of tolvaptan and revealing that retinoic signalling activation is highly effective in slowing cyst growth in organoids and also in a mouse model of ADPKD [139]. It is unclear, however, whether long-term maturation also enables cyst formation in *PKD1^+/-^* and patient-derived CD organoids.

A recent report by our group [140] details a rapid, efficient, and easily applicable method to generate hPSC-derived tubular epithelial organoids in a suspension culture without the addition of ECM. Remarkably, *PKD1^-/-^* organoids spontaneously formed large cystic tubules with high efficiency, also demonstrating cilia defects in cysts, similar to previous reports [126,133]. This platform could therefore serve as a suitable tool for studying cyst formation, as well as drug screening [140].

## 9. Limitations and Potential Advancements of Organoid Models

**Figure 2:**
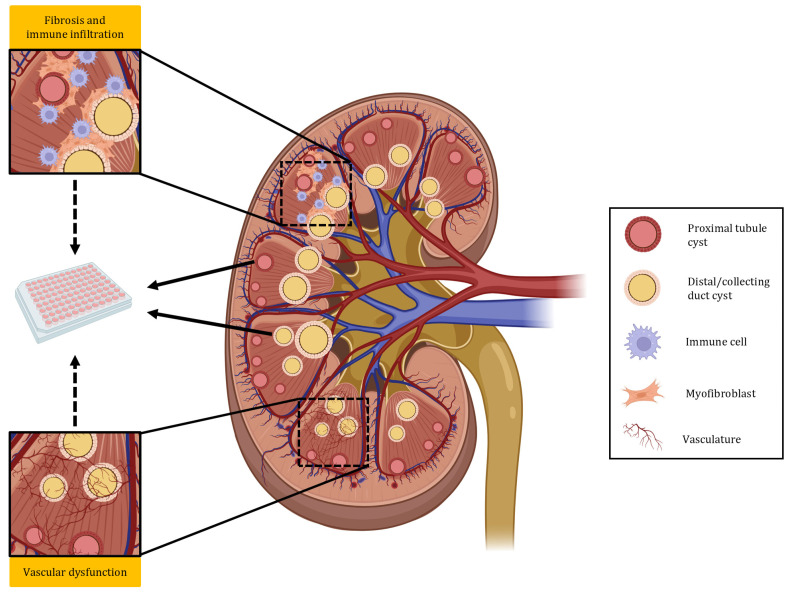
Key pathological features of ADPKD and in vitro modelling. hPSC-derived kidney organoids have been used extensively to study ADPKD; however, so far, only cyst formation in proximal and distal tubules/collecting ducts has been recapitulated. To gain a better understanding of human ADPKD, hPSC-derived kidney organoids should be used to study fibrosis, immune cell infiltration, and vascular dysfunction as they are key features that play important roles in disease progression. Figure generated using Biorender.

Despite these very significant advances in studying ADPKD with complex hPSC-derived renal organoids, a number of limitations remain to be addressed. While many of these organoids have shown great promise in modelling the tubular pathology (shown in Figure 2), none of them appear to contain a well-defined stromal component [124,126,131,132,133,139]. Although the main hallmark of ADPKD is the formation of epithelial cysts, extensive interstitial fibrosis occurs concurrently (shown in Figure 2), contributing significantly to the loss of renal function [141]. There is in fact evidence suggesting that the extent of renal fibrosis may be a better surrogate for renal function decline [142,143], and many ongoing studies are focussed on targeting fibrosis [144]. In CKD, studies have shown that the vast majority of myofibroblasts contributing to fibrosis are derived from resident fibroblasts or invade from the bone marrow [145], indicating that in order to model fibrosis in ADPKD, organoid models would likely necessitate a bona fide stromal component. Currently, two groups have generated kidney organoids from mouse ESCs containing bona fide stromal progenitors, thus recapitulating the higher-order structure [119,146]. Another group has generated Forkhead box D1^+^ (FOXD1^+^) interstitial progenitor-like cells from hPSCs capable of differentiating into mesangial and erythropoietin-producing cells; however, the higher-order kidney structure was not recapitulated by aggregating hPSC-derived UB, MM, and interstitial progenitors [147]. So far, however, kidney organoids with a stromal compartment have not been used to study ADPKD.

Endothelial dysfunction in ADPKD is well documented in patients [148] (shown in Figure 2), as well as in numerous animal models [149], and cardiovascular disease is the leading cause of death in patients [15,16]. Recently endothelium-specific mutations in *Pkd1* and *Pkd2* were shown to independently cause endothelial dysfunction in mice [150,151]. Furthermore, a preprint of a study indicates that in ADPKD mice, the pericystic endothelium contains a specific molecular signature and displays a disorganised microvasculature and that these features are prior to overt renal function loss [152]. This is significant, as the vasculature may be a suitable drug target; however, currently, the renal vasculature has not been studied in kidney organoids.

Macrophages are known to be important in promoting the growth and expansion of ADPKD cysts [153,154] (shown in Figure 2), and the secretion of monocyte chemoattractant protein-1 (MCP-1), a mediator of macrophage infiltration, is associated with early ADPKD [155]. A recent study has established an indirect co-culture method consisting of hPSCs differentiating towards nephron organoids and monocytes or macrophages, showing an important role of secreted extracellular vesicles in promoting cell survival, as well as differentiation [156]. As methods for the co-culture of organoids with immune cells are becoming more widespread [156,157], they are likely to serve as useful tools for uncovering the role of the immune system in conditions such as ADPKD.

The genetics of cystogenesis in ADPKD remains unclear. While patients inherit monoallelic somatic mutations, there is significant evidence indicating that a second hit, be it at the genetic, epigenetic, or post-translational level, may be required to disrupt the function of the residual polycystins. As of now, the majority of organoid models have utilised cells with biallelic mutations in *PKD1* or *PKD2* [124,126,127,130,131,132,133]; however, their physiological relevance is unclear. To date, only two groups have demonstrated cystogenesis in patient hPSC-derived organoids [131,132]. There is some indication that cell maturity and long-term growth can promote cyst formation [139], and monkeys with monoallelic *PKD1* disruptions seem to confirm this [28]. Mosaicism also appears to play a key role, as a small proportion of *Pkd1/PKD1* null renal cells in animal models is sufficient to recapitulate human disease initiation and progression [28,33,34]. Consequently, renal organoid models should investigate more deeply how genetics and long-term cultures impact cystogenesis.

Finally, it is worth considering that the field of hiPSC biology is still relatively new, emerging less than 20 years ago [118]. In the time since, a very large number of cell lines has been developed using different methods and using different tissues of origin. As such, many studies have identified significant variability across hiPSC lines, which can be attributed largely to the donors but also in no small part to individual laboratory-based sources, such as the culture method and passage number used [158]. These sources of variability can therefore significantly affect disease mechanism studies and potentially mask differences between controls and patient-derived cells. Comparisons of hiPSC-derived kidney organoids have also shown that even when using the same differentiation protocols, there is a significant degree of variability between cell lines and different batches in terms of cell maturation, as well as the cell composition of the resulting organoids [159,160]. Specifically, in ADPKD modelling, this variability was shown to have important implications by Mae and colleagues [139], where organoids from independent hiPSC lines showed cystogenesis at timepoints that varied by up to 4 weeks. Importantly, different protocols generate organoids with important differences in terms of cell maturity and composition [161], which has important implications for ADPKD modelling with separate protocols [124,133]. It is also worth mentioning that the reproducibility of a particular organoid differentiation protocol between laboratories must be addressed, as they are known to require cell line-dependent optimisation [121,133], and the causes remain as of yet unknown. While using different hiPSC genotypes in modelling human disease is important to capture the variability between individuals, reference “gold-standard” cell lines with known characteristics, morphologies, and differentiation patterns may help to address the issue of variability and reproducibility [158].

## 10. Conclusions

Decades of ADPKD research have provided researchers with valuable insights into the genetics and the pathogenesis of renal cysts; however, so far, these have not yielded adequate treatments. While animal studies have uncovered important aspects of ADPKD, they are not readily translatable to the human setting. Kidney organoids derived from hPSCs have seen considerable advances in the last 10 years, recapitulating numerous aspects of kidney development and architecture. These organoids have also been used to model cystogenesis in ADPKD, uncovering new insights into disease initiation and progression, with some having been applied in the high-throughput screening of novel drug compounds and having yielded potentially effective candidates. Despite this, limitations remain; aspects such as inflammation and fibrosis, which are clinically relevant in disease progression, have not yet been modelled, indicating that further improvements are required.
