# Correction: Scarlat et al. Autosomal Dominant Polycystic Kidney Disease: From Pathogenesis to Organoid Disease Models. *Biomedicines* 2025, *13*, 1766

**DOI:** 10.3390/biomedicines13123059

**Published:** 2025-12-12

**Authors:** Alexandru Scarlat, Susanna Tomasoni, Piera Trionfini

**Affiliations:** Istituto di Ricerche Farmacologiche Mario Negri IRCCS, Centro Anna Maria Astori, Science and Technology Park Kilometro Rosso, 24126 Bergamo, Italy

## Text Correction

In the present article [1] the study originally cited as Ref. 51 (Spirli, C.; Locatelli, L.; Fiorotto, R.; Morell, C.M.; Fabris, L.; Pozzan, T.; Strazzabosco, M. Altered Store Operated Calcium Entry Increases cAMP production and ERK1/2 phosphorylation in Polycystin-2 Defective Cholangiocytes. *Hepatology* **2012**, *55*, 856–868.) has been retracted, and the citation has been removed accordingly. Consequently, the phrase referencing the article in question “…the depletion of calcium in the endoplasmic reticulum indirectly activates AC6.” (in Section 3.1) has also been removed.

With this correction, the order of some references has been adjusted accordingly. The authors state that the scientific conclusions are unaffected. This correction was approved by the Academic Editor. The original publication has also been updated.
